# Upregulating carnitine palmitoyltransferase 1 attenuates hyperoxia-induced endothelial cell dysfunction and persistent lung injury

**DOI:** 10.1186/s12931-022-02135-1

**Published:** 2022-08-13

**Authors:** Jason L. Chang, Jiannan Gong, Salu Rizal, Abigail L. Peterson, Julia Chang, Chenrui Yao, Phyllis A. Dennery, Hongwei Yao

**Affiliations:** 1 Division of Biology and Medicine, Department of Molecular Biology, Cell Biology and Biochemistry, Brown University, 185 Meeting Street, SFH, Providence, RI 02912 USA; 2 Department of Respiratory Medicine, Second Hospital of Shanxi Medical University, Shanxi Medical University, Taiyuan, Shanxi China; 3 Department of Pediatrics, Warren Alpert Medical School of Brown University, Providence, RI USA

**Keywords:** Bronchopulmonary dysplasia, Fatty acid oxidation, Apoptosis, Migration, Angiogenesis

## Abstract

**Background:**

Bronchopulmonary dysplasia (BPD) is a chronic lung disease in premature infants that may cause long-term lung dysfunction. Accumulating evidence supports the vascular hypothesis of BPD, in which lung endothelial cell dysfunction drives this disease. We recently reported that endothelial carnitine palmitoyltransferase 1a (Cpt1a) is reduced by hyperoxia, and that endothelial cell-specific Cpt1a knockout mice are more susceptible to developing hyperoxia-induced injury than wild type mice. Whether Cpt1a upregulation attenuates hyperoxia-induced endothelial cell dysfunction and lung injury remains unknown. We hypothesized that upregulation of Cpt1a by baicalin or l-carnitine ameliorates hyperoxia-induced endothelial cell dysfunction and persistent lung injury.

**Methods:**

Lung endothelial cells or newborn mice (< 12 h old) were treated with baicalin or l-carnitine after hyperoxia (50% and 95% O_2_) followed by air recovery.

**Results:**

We found that incubation with l-carnitine (40 and 80 mg/L) and baicalin (22.5 and 45 mg/L) reduced hyperoxia-induced apoptosis, impaired cell migration and angiogenesis in cultured lung endothelial cells. This was associated with increased Cpt1a gene expression. In mice, neonatal hyperoxia caused persistent alveolar and vascular simplification in a concentration-dependent manner. Treatment with l-carnitine (150 and 300 mg/kg) and baicalin (50 and 100 mg/kg) attenuated neonatal hyperoxia-induced alveolar and vascular simplification in adult mice. These effects were diminished in endothelial cell-specific Cpt1a knockout mice.

**Conclusions:**

Upregulating Cpt1a by baicalin or l-carnitine ameliorates hyperoxia-induced lung endothelial cell dysfunction, and persistent alveolar and vascular simplification. These findings provide potential therapeutic avenues for using l-carnitine and baicalin as Cpt1a upregulators to prevent persistent lung injury in premature infants with BPD.

## Introduction

Ventilatory support such as supplemental oxygen used to save these premature infants may impair the growth of the pulmonary vasculature and of distal alveoli [1]. This leads to bronchopulmonary dysplasia (BPD), which affects 10,000 to 15,000 premature infants annually in the US, and costs more than $225,000 (USD) per infant during the initial hospitalization [2, 3]. Some BPD survivors have pulmonary and cardiovascular sequelae in adolescence and adulthood [4–7]. This is corroborated by animal models of BPD showing impaired lung function, arrested alveolarization, simplified vasculature, and dysmorphic vascular development [8–10]. There are limited therapeutic interventions available for BPD. Pharmacological treatments for BPD include steroids, surfactant, caffeine, and vitamin A [11]. Although these therapies have improved the survival of premature infants, they have minimally reduced BPD prevalence and associated persistent lung injury [1, 12]. Thus, it is urgent to develop new therapies for preventing the persistent lung injury seen in BPD.

Although cross-talk between epithelial and endothelial cells plays important roles in lung injury and repair, accumulating evidence supports the vascular hypothesis of BPD, in which lung endothelial cell dysfunction drive the disease [13–15]. Therefore, targeting dysfunctional endothelial cells could provide new therapeutic options for BPD. Endothelial cells primarily rely on glycolysis for bioenergetics. Both glycolysis and mitochondrial fatty acid oxidation (FAO) play important roles in modulating endothelial cell proliferation and vessel sprouting [16, 17]. We previously reported that hyperoxic exposure decreased FAO, leading to apoptosis in lung endothelial cells [10]. Carnitine palmitoyltransferase 1 (Cpt1) is the rate-limiting enzyme of the carnitine shuttle responsible for transporting long-chain fatty acids into mitochondria for β-oxidation during the FAO. This enzyme has three isoforms: Cpt1a, Cpt1b and Cpt1c. While only Cpt1a and Cpt1b have acyltransferase activity, Cpt1a has ten-fold higher affinity for carnitine than Cpt1b to generate acylcarnitine for mitochondrial transport of long-chain fatty acids [18]. We have further demonstrated that endothelial cell-specific Cpt1a knockout mice were susceptible to developing hyperoxia-induced alveolar and vascular simplification compared to wild type littermates as neonates [10]. Nevertheless, whether upregulating Cpt1a ameliorates neonatal hyperoxia-induced persistent lung injury remains unclear.

l-Carnitine is a substrate of Cpt1 that transfers activated long-chain fatty acids across the inner mitochondrial membrane for β-oxidation [19]. l-carnitine also increases Cpt1 gene, protein and enzyme activity, which is associated with activation of the peroxisome proliferator activated receptor α (PPARα), PPARγ, and PPARγ coactivator (PGC)-1α [10, 20–22]. A recent study has shown that baicalin, a major flavonoid component from the herbal medicine *Scutellaria baicalensis*, can directly bind to, and activate Cpt1a in the liver [23]. Therefore, we hypothesized that upregulating Cpt1a by l-carnitine or baicalin protects against hyperoxia-induced endothelial cell dysfunction and persistent lung injury. To test this hypothesis, lung endothelial cells and neonatal mice (< 12 h old) were exposed to hyperoxia (50% and 95% O_2_) followed by air recovery. We then treated them with l-carnitine or baicalin, and determined apoptosis, proliferation, migration and angiogenesis in vitro, as well as lung function, alveolar and vascular simplification in mice in vivo.

## Methods

### Cell culture and treatments

Mouse fetal lung endothelial cell lines (MFLM-91U cells) were purchased from Seven Hills Bioreagents (Cincinnati, Ohio), and cultured in UltraCulture medium (Biowhittaker) [10]. There was no mycoplasma contamination in these cells. Cells were incubated with l-carnitine (40 and 80 mg/L), or baicalin (22.5 and 45 mg/L) for 12 h during the air recovery phase after hyperoxic exposure. At these concentrations, l-carnitine increased FAO in lung endothelial cells [10], while baicalin upregulated Cpt1a gene expression and enzymatic activity in Hela cells without affecting cell viability [23].

### Hyperoxic exposure

Cells at 70–80% confluence were exposed to hyperoxia (50% O_2_/5% CO_2_, 95% O_2_/5% CO_2_) or air (21% O_2_/5% CO_2_) for 24 h followed by normoxia (21% O_2_/5% CO_2_) for 24 h [10]. Culture media were changed every 24 h. Newborn mice (< 12 h old, male and female) along with their mothers were exposed to room air (21% O_2_) or hyperoxia (50% O_2_ or 95% O_2_) for 72 h in an A-chamber (BioSpherix, Redfield, NY) [10]. The dams were switched every 24 h between room air and hyperoxia to avoid injury. The pups were allowed to recover in room air until postnatal day (pnd)14 or pnd60.

### Administration of l-carnitine or baicalin in mice

Lung alveolar formation starts at pnd4, and peaks between postnatal day (pnd) 10 to pnd14 [24, 25]. Thus, l-carnitine (150 and 300 mg/kg) [10] or baicalin (50 and 100 mg/kg) [23] was administered daily through intraperitoneal injection (i.p.) to mice for 10 days (between pnd4 and pnd13), after they were exposed to hyperoxia for 3 days as neonates. At pnd14 and pnd60, mice were sacrificed, and lungs were used for further analysis.

### Measurement of steady-state mRNA levels

We extracted total RNA by the TRIzol reagent, and purified them using the RNeasy miniprep kit (Qiagen, Valencia, CA). RNA samples were quantified by the NanoDrop One Microvolume UV–Vis Spectrophotometer (Thermo Fisher Scientific, Waltham, MA). A total of 400 nanograms of RNA were used for reverse transcription with the Taqman Reverse Transcription Reagents (Thermo Fisher Scientific). One microliter of cDNA was used for real-time PCR reactions by the 7300 Real-Time PCR System (Applied Biosystems, Waltham, MA). All Taqman gene probes (Cpt1a, Cat#: Mm01231183; Cpt1b, Cat#: Mm00487191; Cpt1c, Cat#: Mm00463970; 18 s, Cat#: Hs99999901_s1) were purchased from Thermo Fisher Scientific. Gene expression was normalized to 18 s rRNA levels. Relative RNA abundance was quantified by the comparative 2^−ΔΔCt^ method.

### Detection of FAO

Mitochondrial utilization of fatty acids was measured by the Seahorse XF Analyzer (Seahorse Bioscience, North Billerica, MA) according to the instructions for the Seahorse XF Mito Fuel Flex Test Assay [10]. Briefly, oxygen consumption rate (OCR) was measured after sequential injection of these inhibitors: etomoxir, a specific Cpt1 inhibitor, 4 μM; UK5099, a mitochondrial pyruvate carrier inhibitor, 2 μM; BPTES, a selective glutaminase inhibitor, 3 μM and after optimization. The FAO measures reliance on fatty acid utilization to maintain baseline respiration, which was calculated using the formula: dependency (%) = (baseline OCR -target OCR)/(baseline OCR- all inhibitors OCR) × 100.

### Apoptosis assessment by flow cytometric assay

The cells were washed once with cold phosphate buffered saline (PBS), and resuspended in Annexin V binding buffer. The 100 μl Annexin V binding buffer containing 100,000–200,000 cells was incubated with 5 μl of FITC conjugated Annexin-V and 5 μl of PI (Life Technologies, Carlsbad, CA), at room temperature for 15 min. After the incubation period, 400 μl of Annexin V binding buffer was added and gently mixed, and then analyzed using the BD FACSAria (BD Bioscience, Woburn, MA). Annexin V positive cells were expressed as a simple percentage of 20,000 events analyzed [10].

### Caspase-3 activity assay

Cellular caspase-3 activity was measured according to the caspase-3 colorimetric assay kit (BioVision, Milpitas, CA) [10]. In brief, cell pellets were lysed in the chilled cell lysis buffer. Cytosolic proteins (100–200 μg) were reacted with DEVD-*p*-nitroanilide substrate in reaction buffer containing 10 mM dithiothreitol to determine caspase-3 activity. The *p*-nitroanilide light emission was quantified using a Benchmark microplate reader (Bio-Rad) at 405 nm.

### Evaluation of cell proliferation

In vitro proliferation was determined by Click-iT EdU Flow Cytometry Assay Kit from Invitrogen according to the manufacturer’s protocol [10]. Briefly, cells were incubated with 10 μM of a nucleoside analog of thymidine (5-ethynyl-2′deoxyuridine, EdU) for 2 h. After washing and fixation, incorporated EdU during DNA synthesis was labeled with Alexa Fluor 488 azide in the provided reaction buffer for 30 min. Cells were analyzed using the BD FACSAria (BD Bioscience).

For EdU detection in the lung, mice were intraperitoneally injected with EdU (50 mg/kg, daily) for 3 days, and then sacrificed at 24 h after the last EdU injection. Click-iT EdU Cell Proliferation Kit for Imaging, Alexa Fluor 647 was used to determine proliferation in lungs, which was co-staining with the endothelial cell marker von Wildebrand factor (vWF) [10]. The numbers of EdU-positive cells were counted in 3 randomly selected high-power fields (HPF) in a blinded manner using a Zeiss Axiovert 200 M Fluorescence Microscope.

### Determination of cell migration

Migration was assessed in cells using the Scratch assay [8]. Once reaching ~ 90% confluence, the cells in the culture plates was scratched using a 200 µl pipette tip. Cells were then washed with PBS and cultured in medium containing 0.1% FBS, mitomycin C (20 µm), and VEGF (50 ng/ml) for 16 h. Images were captured at × 10 magnification on a Zeiss Axiovert 200 M Microscope. The size of the denuded area was assessed to negatively reflect migration using MiToBo analyzer software in Image J.

### Endothelial tube formation assay

An in vitro angiogenesis assay was performed using the Endothelial Tube Formation assay kit (Cell Biolab Inc., San Diego, CA) according to the manufacturer’s protocol. Briefly, ECM gel was thawed in a frost-free 4 °C refrigerator overnight, and added into a pre-chilled 96-well sterile plate. An ECM gel was formed when this plate was incubated 1 h at 37 °C. After hyperoxia for 24 h, cells (2 × 10^4^ cells/well) were seeded on a plate precoated with ECM gel, and incubated with l-carnitine or baicalin for 4 h under normoxic conditions. Capillary-like tubes were photographed under a microscope, and all side branches were counted using the Angiogenesis Analyzer plug-in and ImageJ software.

### Immunofluorescence

Lung sections were de-paraffinized and subjected to heat-mediated antigen retrieval in a citrate buffer solution (Vector Labs). Immunofluorescence was performed to determine apoptosis with TUNEL staining in lungs overnight at 4 °C with or without an antibody against vWF (1:100 dilution, A0082, Dako) [26]. After incubation with secondary antibody (1:5000 dilution) for 1 h at room temperature, sections were mounted in hard-set mounting medium containing DAPI (Vector Labs) and allowed to incubate overnight. Numbers of TUNEL^+^/vWF^+^ or vWF^+^ vessels were counted in 3 randomly selected high power filed (HPF) using a Zeiss Axiovert 200 M Fluorescence Microscope. These experiments were carried out in a blinded manner.

### Evaluating lung morphometry

Mean linear intercept (Lm) and radial alveolar count (RAC) were measured in mouse lungs stained with hematoxylin and eosin (H&E) as previously described [10]. In brief, we inflated non-lavaged mouse lungs with 1% low melt agarose at a pressure of 25 cm H_2_O, and fixed them with 4% neutral buffered paraformaldehyde. These fixed lungs were embedded in paraffin, and sectioned into 4 µm sections using a rotary microtome (MICROM International GmbH, Walldorf, Germany). Lung midsagittal sections with H&E staining were utilized to determine airspace Lm. A perpendicular line was drawn from the center of the respiratory bronchiole to the distal acinus (as defined by the pleura or the nearest connective tissue septum). The number of septae intersected by each line was counted as RAC, and a minimum of 8 counts were performed for each animal.

### Statistical analysis

The results were expressed as mean ± SEM. Statistical analyses were performed using GraphPad Prism 9 in a blinded manner wherever possible. The statistical significance of the differences was evaluated by using one-way ANOVA followed by Tukey’s post-test to specifically compare indicated groups during multiple comparisons. For in vitro studies, statistical analysis was performed on at least three biological replicates. For in vivo studies, sample size was at least five animals per group. Statistical significance was considered at a *P* value of < 0.05 vs controls.

## Results

### Both l-carnitine and baicalin restore Cpt1a gene expression and FAO in cultured lung endothelial cells exposed to hyperoxia

**Fig. 1:**
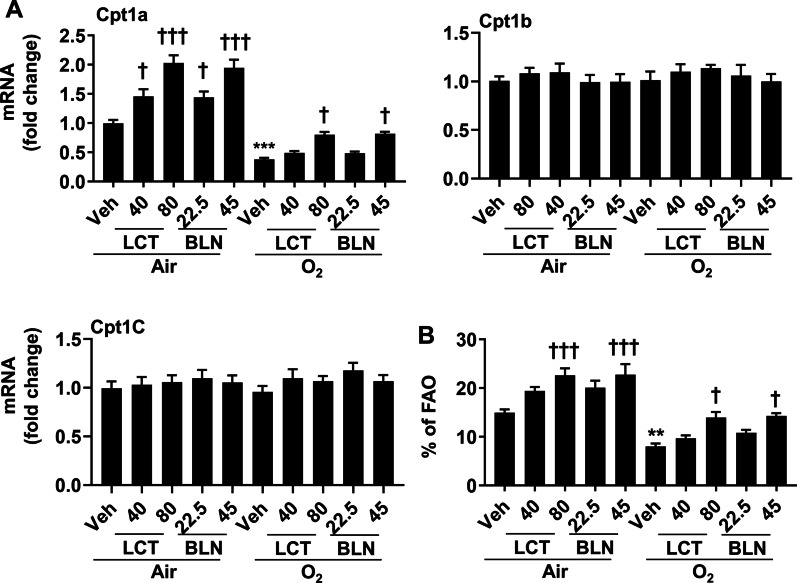
Incubation with l-carnitine and baicalin restored Cpt1a gene expression and FAO in cultured lung endothelial cells exposed to hyperoxia. Cells were exposed to hyperoxia (95% O_2_/5% CO_2_) or air (21% O_2_/5% CO_2_) for 24 h followed by normoxia for 24 h. Cells were incubated with l-carnitine (LCT, 40 and 80 mg/L) and baicalin (BLN, 22.5 and 45 mg/L) for 12 h during the air recovery phase. Expression of Cpt1a, Cpt1b, and Cpt1c genes was measured by qRT-PCR, while the FAO was measured by the Seahorse Analyzer. N = 6. **P < 0.01, ***P < 0.001 vs veh/air. ^†^*P* < 0.05, ^†††^*P* < 0.001 vs corresponding veh group using one-way ANOVA followed by Tukey’s multiple comparison test

We have reported that hyperoxic exposure decreased Cpt1a and FAO in lung endothelial cells [10]. To determine whether l-carnitine and baicalin attenuate hyperoxia-induced reduction of Cpt1 gene expression, we first assessed cell viability using the Trypan blue exclusion assay after incubation with l-carnitine or baicalin for 12 h in MFLM-91U cells under normoxic conditions. Cell viability was 100%, 98.4%, 99.1%, 99.2%, 98.3% in vehicle, l-carnitine (40 mg/L), l-carnitine 80 mg/L), baicalin (22.5 mg/L), baicalin (45 mg/L) groups, respectively. We then detected the expression of Cpt1a, Cpt1b and Cpt1c genes by qRT-PCR in hyperoxia-exposed MFLM-91U cells treated with l-carnitine (40 and 80 mg/L) or baicalin (22.5 and 45 mg/L) for 12 h. As expected, hyperoxia (95% O_2_/5% CO_2_) significantly decreased Cpt1a but not Cpt1b or Cpt1c gene expression [10] (Fig. 1A). Incubation with l-carnitine (40 and 80 mg/L) and baicalin (22.5 and 45 mg/L) significantly increased Cpt1a gene expression in both normoxic and hyperoxic conditions, in a concentration-dependent manner (Fig. 1A). There were no changes in Cpt1b or Cpt1c gene expression in cells treated with l-carnitine or baicalin at any concentration.

We further measured FAO using the Seahorse Analyzer in hyperoxia-exposed cells treated with l-carnitine (40 and 80 mg/L) or baicalin (22.5 and 45 mg/L) for 12 h. As shown in Fig. 1B, hyperoxic exposure (95% O_2_/5% CO_2_) decreased the levels of FAO. l-carnitine and baicalin significantly restored FAO in both normoxic and hyperoxic conditions, in a concentration-dependent manner (Fig. 1B). These data demonstrate that both l-carnitine and baicalin attenuate hyperoxia-induced reduction of Cpt1a gene expression and FAO in cultured lung endothelial cells.

### Incubation with l-carnitine and baicalin ameliorates hyperoxia-induced apoptosis in cultured lung endothelial cells

**Fig. 2:**
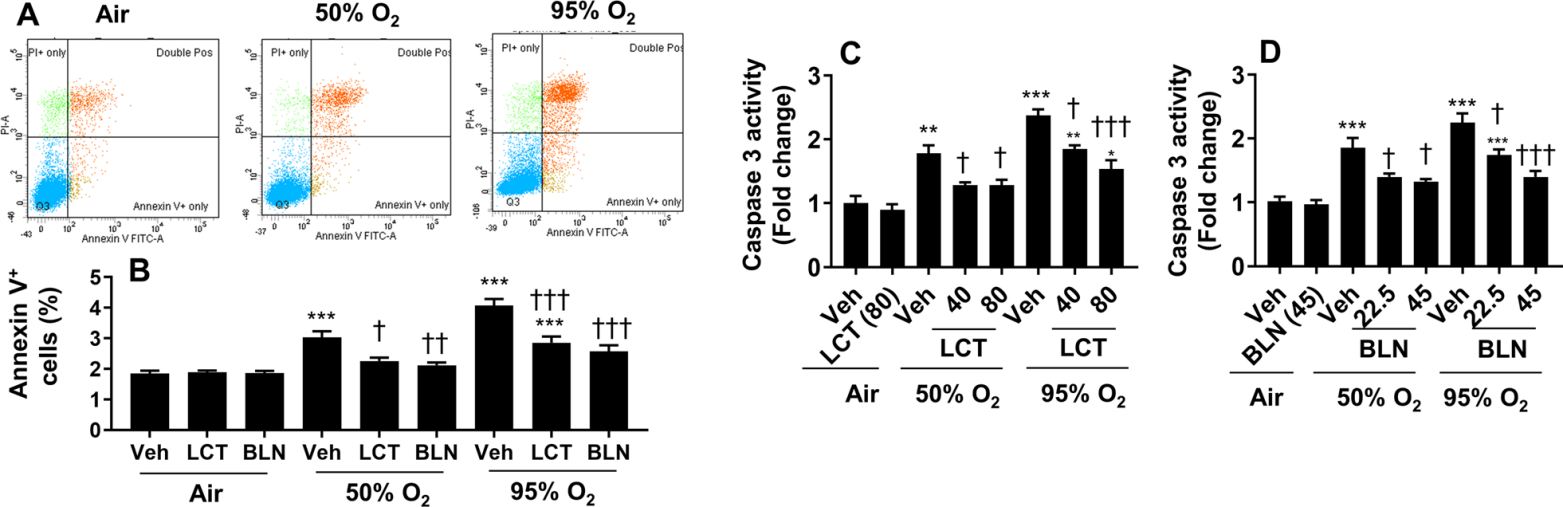
Incubation with l-carnitine and baicalin attenuated hyperoxia-induced apoptosis in cultured lung endothelial cells. Cells were exposed to hyperoxia (50% O_2_/5% CO_2_, 95% O_2_/5% CO_2_) or air (21% O_2_/5% CO_2_) for 24 h followed by normoxia for 24 h. Cells were incubated with l-carnitine (LCT, 40 and 80 mg/L) and baicalin (BLN, 22.5 and 45 mg/L) for 12 h during the air recovery phase. In panel B, LCT (80 mg/L) and BLN (45 mg/L) were used. Apoptosis was detected by measuring Annexin V^+^ cells via flow cytometry and caspase 3 activity using a kit. N = 4–8. **P < 0.01, ***P < 0.001 vs veh/air. ^†^*P* < 0.05, ^††^*P* < 0.01, ^†††^*P* < 0.001 vs corresponding O_2_/veh group using one-way ANOVA followed by Tukey’s multiple comparison test

Hyperoxic exposure causes apoptosis in lung endothelial cells [10, 27, 28]. Here, we determined whether upregulating Cpt1a by l-carnitine or baicalin ameliorates hyperoxia-induced apoptosis. As shown in Fig. 2A, hyperoxic exposure (50% O_2_/5% CO_2_ or 95% O_2_/5% CO_2_) increased the number of annexin V^+^ cells in an oxygen concentration-dependent manner. These effects were significantly reduced by both l-carnitine (40 and 80 mg/L) and baicalin (22.5 and 45 mg/L) incubation (Fig. 2B). Similarly, caspase-3 activity was increased in cells exposed to hyperoxia in an oxygen concentration dependent manner, and this was reduced by l-carnitine (40 and 80 mg/L) and baicalin (22.5 and 45 mg/L) incubation (Fig. 2C, D). These results suggest that l-carnitine or baicalin attenuates hyperoxia-induced apoptosis in cultured lung endothelial cells.

### Both l-carnitine and baicalin protect against hyperoxia-induced repression of migration in cultured lung endothelial cells

**Fig. 3:**
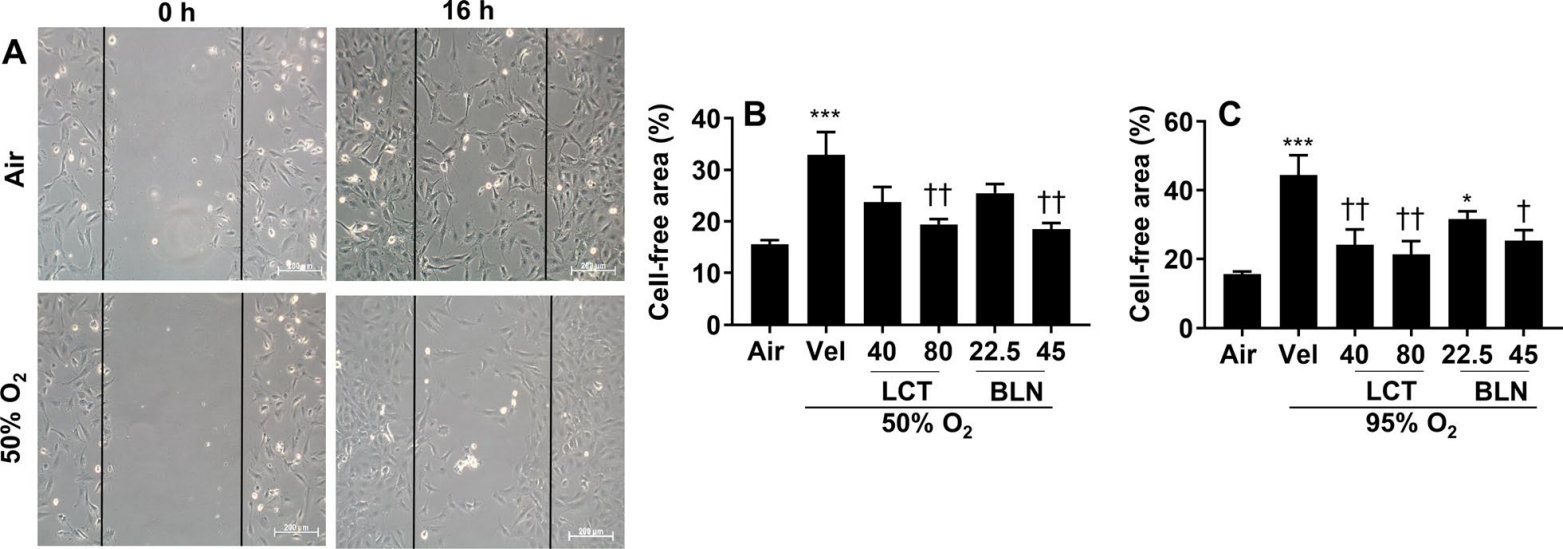
Incubation with l-carnitine and baicalin restored hyperoxia-induced reduction of migration in cultured lung endothelial cells. Cells were exposed to hyperoxia (50% O_2_/5% CO_2_, 95% O_2_/5% CO_2_) or air (21% O_2_/5% CO_2_) for 24 h followed by normoxia for 16 h. Cells were incubated with l-carnitine (LCT, 40 and 80 mg/L) and baicalin (BLN, 22.5 and 45 mg/L) for 12 h during the air recovery phase. Migration was measured using scratch assay. N = 6. *P < 0.05, ***P < 0.001 vs air. ^†^*P* < 0.05, ^††^*P* < 0.01, vs corresponding O_2_/veh group using one-way ANOVA followed by Tukey's multiple comparison test

We and other have shown that hyperoxic exposure reduces endothelial cell migration [8, 29]. It is not clear whether upregulating Cpt1a protects against hyperoxia-induced reduction in endothelial cell migration. A scratch assay was performed in cells 16 h after hyperoxic exposure to measure cell migration. As expected, hyperoxia (50% O_2_/5% CO_2_ and 95% O_2_/5% CO_2_) significantly reduced cell migration in an oxygen concentration-dependent manner (Fig. 3A–C). These effects were significantly attenuated by both l-carnitine (40 and 80 mg/L) and baicalin (22.5 and 45 mg/L) incubation (Fig. 3B, C). These data demonstrate that hyperoxia-induced repression of cell migration is attenuated by l-carnitine or baicalin.

### Incubation with l-carnitine and baicalin has no effect on hyperoxia-induced proliferation in cultured lung endothelial cells

**Fig. 4:**
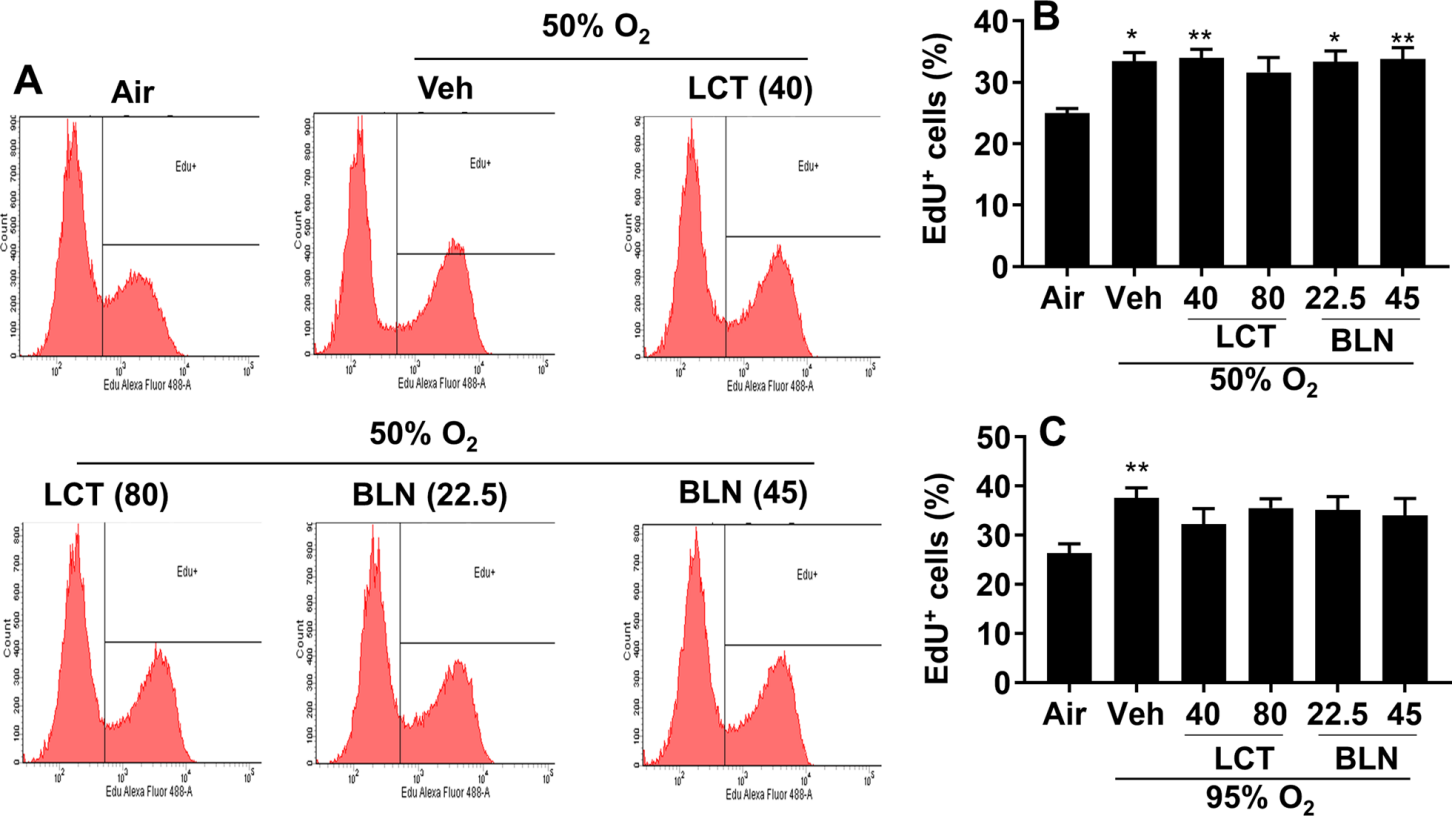
Incubation with l-carnitine and baicalin did not affect hyperoxia-induced proliferation in cultured lung endothelial cells. Cells were exposed to hyperoxia (50% O_2_/5% CO_2_, 95% O_2_/5% CO_2_) or air (21% O_2_/5% CO_2_) for 24 h followed by normoxia for 24 h. Cells were incubated with l-carnitine (LCT, 40 and 80 mg/L) and baicalin (BLN, 22.5 and 45 mg/L) for 12 h during the air recovery phase. EdU incorporation was measured by flow cytometry, and a percentage of EdU positive cells was calculated. N = 6–7. *P < 0.05, **P < 0.01 vs air using one-way ANOVA followed by Tukey’s multiple comparison test

We recently reported that hyperoxia followed by air recovery increased proliferation in cultured lung endothelial cells [8]. To determine the effects of l-carnitine or baicalin on hyperoxia-induced proliferation, we evaluated proliferation using the Click-iT EdU Cell Proliferation Kit. As expected, hyperoxia (50% O_2_/5% CO_2_ and 95% O_2_/5% CO_2_) significantly increased EdU incorporation in cultured lung endothelial cells (Fig. 4). There were no effects of l-carnitine (40 and 80 mg/L) or baicalin (22.5 and 45 mg/L) on hyperoxia-induced EdU incorporation (Fig. 4B, C). Hence, l-carnitine or baicalin treatment has no effect on hyperoxia-induced proliferation in cultured lung endothelial cells.

### Incubation with l-carnitine and baicalin attenuates hyperoxia-induced disruption of angiogenesis in vitro

**Fig. 5:**
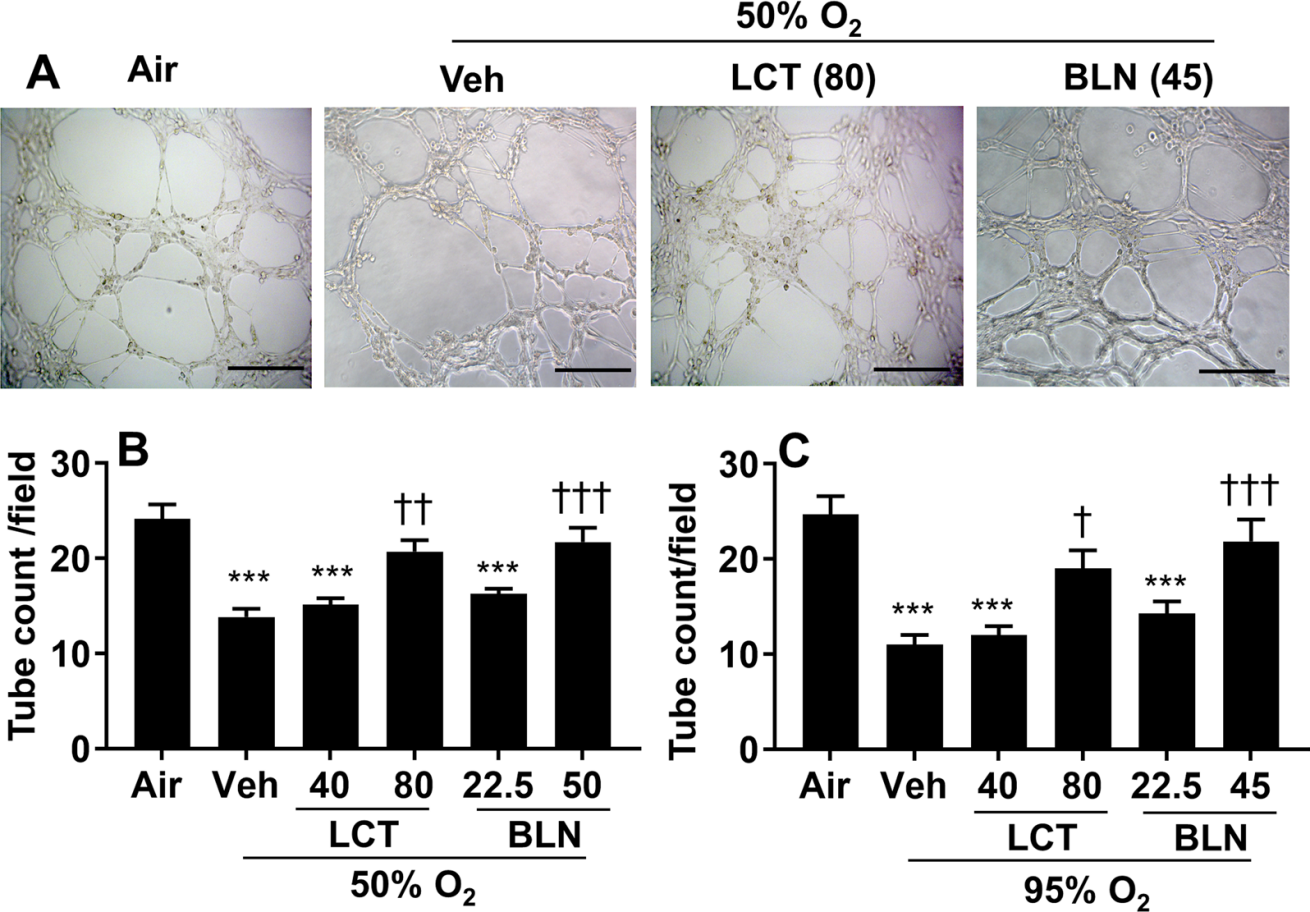
Incubation with l-carnitine and baicalin attenuated hyperoxia-induced impairment of tube formation in cultured lung endothelial cells. Cells were exposed to hyperoxia (50% O_2_/5% CO_2_, 95% O_2_/5% CO_2_) for 24 h. Cells were then seeded on a plated precoated with ECM gel and treated with l-carnitine (LCT, 40 and 80 mg/L) and baicalin (BLN, 22.5 and 45 mg/L) for 4 h under normoxic conditions. Scale bar: 100 μm. N = 7. ***P < 0.001 vs air. ^†^*P* < 0.05, ^††^*P* < 0.01, ^†††^*P* < 0.001 vs corresponding O_2_/veh group using one-way ANOVA followed by Tukey’s multiple comparison test

To determine the effect of hyperoxia on angiogenesis in vitro, we performed tube formation assays. As shown in Fig. 5A, hyperoxia (50% O_2_/5% CO_2_ and 95% O_2_/5% CO_2_) disrupted vascular tube formation on Matrigel in an oxygen concentration-dependent manner. These effects were attenuated by both l-carnitine (80 mg/L) and baicalin (45 mg/L) incubation during the air recovery phase (Fig. 5B, C). Therefore, l-carnitine or baicalin incubation protects against hyperoxia-induced disruption of angiogenesis in vitro.

### Treatment with l-carnitine and baicalin protects against hyperoxia-induced lung function decline

**Fig. 6:**
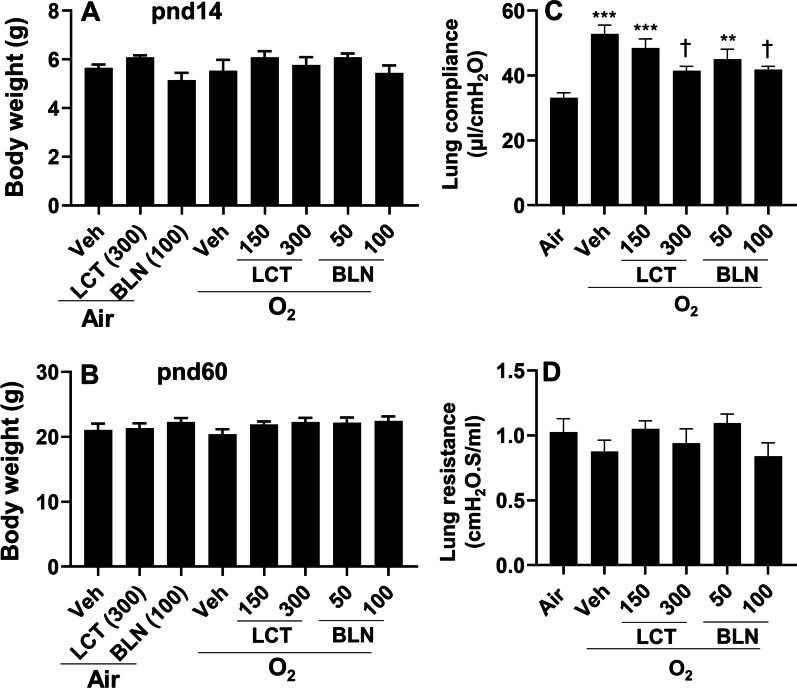
Treatment with l-carnitine and baicalin attenuated neonatal hyperoxia-induced lung function decline in adult mice. C57BL/6 J mice (< 12 h old) were exposed to 95% O_2_ for 3 days, and allowed to recover in air until pnd14 and pnd60. Mice were treated with l-carnitine (LCT, 150 and 300 mg/kg, i.p.) and baicalin (BLN, 50 and 100 mg/kg, i.p.) daily between pnd4 and pnd13. Body weight were assessed at both pnd14 and pnd60 (**A**, **B**), while lung compliance and resistance were evaluated using the FlexiVent system at pnd60 (**C**, **D**). N = 5–10. **P < 0.01, ***P < 0.001 vs air. ^†^*P* < 0.05, vs O_2_/veh group using one-way ANOVA followed by Tukey’s multiple comparison test

To determine whether our in vitro findings could be replicated in an in vivo mouse model, neonatal mice (< 12 h old) were exposed to hyperoxia (95% O_2_) for 3 days and allowed to recover in room air until postnatal day (pnd)14 or pnd60. These mice were treated daily with l-carnitine (150 and 300 mg/kg, i.p.) and baicalin (50 and 100 mg/kg, i.p.) between pnd4 and pnd13. As shown in Fig. 6A, B, neither neonatal hyperoxia nor drug treatments altered body weight at both pnd14 and pnd60. Neonatal hyperoxia adversely affects lung function in adult mice [26]. Thus, we wanted to evaluate whether l-carnitine or baicalin treatment attenuates neonatal hyperoxia-induced lung function decline in adult mice using the FlexiVent system. As expected, neonatal hyperoxia (95% O_2_) increased lung compliance at pnd60 (Fig. 6C). This effect was significantly reduced by both l-carnitine (300 mg/kg) and baicalin (100 mg/kg) treatment (Fig. 6C). Neither neonatal hyperoxia nor l-carnitine or baicalin treatment altered lung resistance (Fig. 6D). These results demonstrate that l-carnitine or baicalin attenuates neonatal hyperoxia-induced lung function decline in adult mice.

### Treatment with l-carnitine and baicalin does not affect proliferation but attenuates apoptosis in the lung of mice exposed to hyperoxia as neonates

**Fig. 7:**
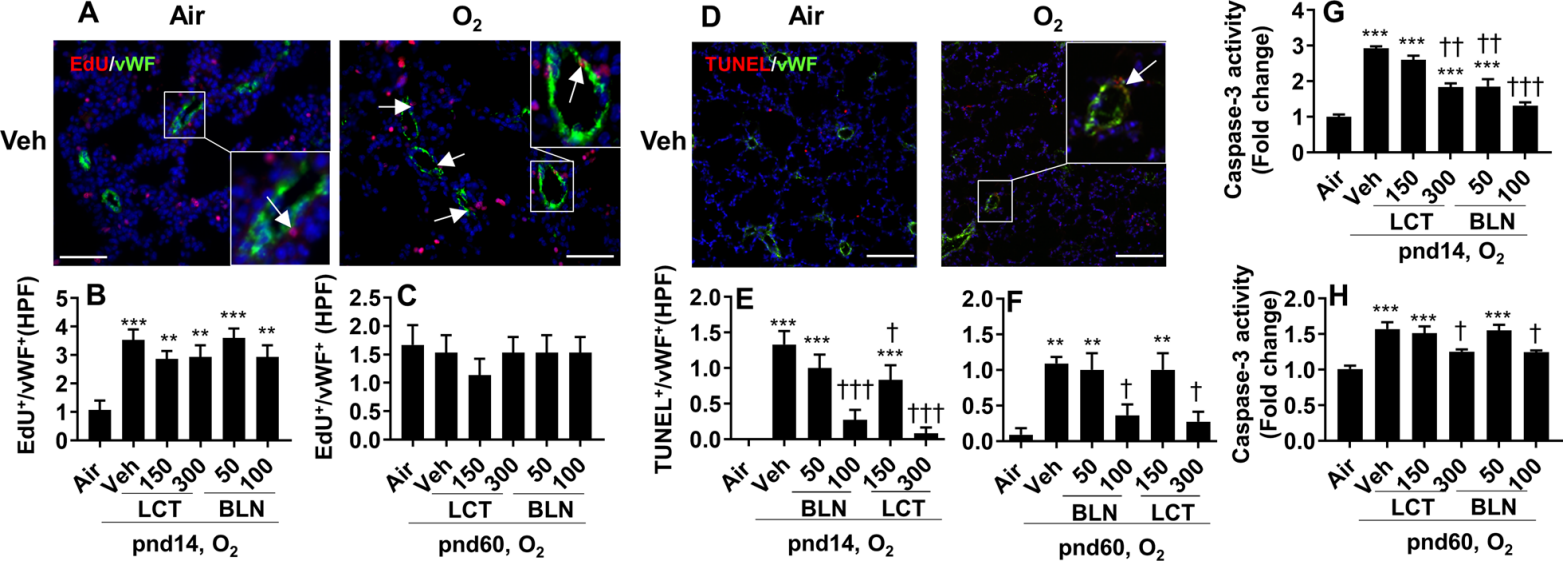
Treatment with l-carnitine and baicalin attenuated neonatal hyperoxia-induced apoptosis in the lung. C57BL/6 J mice (< 12 h old) were exposed to 95% O_2_ for 3 days, and allowed to recover in air until pnd14 and pnd60. Mice were treated with l-carnitine (LCT, 150 and 300 mg/kg, i.p.) and baicalin (BLN, 50 and 100 mg/kg, i.p.) daily between pnd4 and pnd13. EdU incorporation (**A**–**C**), TUNEL staining (**D**–**F**) and caspase-3 activity (**G**, **H**) were evaluated in the lung. Scale bar: 100 μm. N = 5–6. **P < 0.01, ***P < 0.001 vs air. ^†^*P* < 0.05, ^††^*P* < 0.01, ^†††^*P* < 0.001 vs O_2_/veh group using one-way ANOVA followed by Tukey’s multiple comparison test

We recently reported that hyperoxic exposure increased lung endothelial cell proliferation in neonatal mice [8]. Here, we evaluated the effects of l-carnitine and baicalin on neonatal hyperoxia-induced endothelial cell proliferation in the lung. As shown in Fig. 7A, B, EdU incorporation was increased in lung endothelial cells of mice exposed to hyperoxia at pnd14. At pnd60, there were no changes in EdU incorporation in lung endothelial cells between air and hyperoxia-exposed mice (Fig. 7C). These effects were not affected by l-carnitine or baicalin treatment at pnd14 or pnd60 (Fig. 7B, C).

We next assessed the effects of l-carnitine or baicalin treatment on hyperoxia-induced apoptosis in lung endothelial cells of mice. As shown in Fig. 7D–F, neonatal hyperoxia significantly increased number of TUNEL^+^ endothelial cells in mouse lungs at both pnd14 and pnd60. These effects were significantly reduced by both l-carnitine and baicalin treatment (Fig. 7E, F). Similarly, l-carnitine or baicalin treatment significantly attenuated the neonatal hyperoxia-induced increase in caspase-3 activity in the lung at both pnd14 and pnd60 (Fig. 7G, H). Altogether, these data demonstrate that l-carnitine or baicalin treatment does not affect proliferation but ameliorates apoptosis in the lung of mice exposed to hyperoxia as neonates.

### Treatment with l-carnitine and baicalin ameliorates neonatal hyperoxia-induced persistent alveolar and vascular simplification

**Fig. 8:**
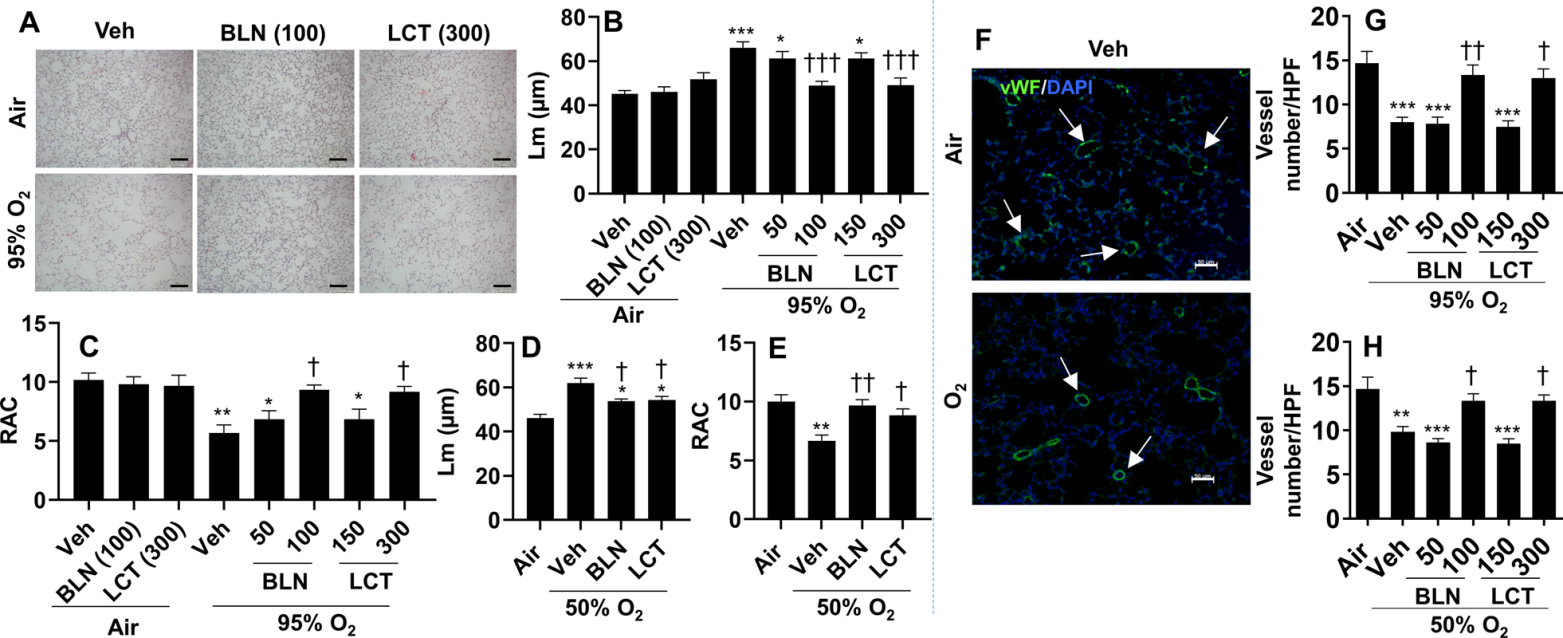
Treatment with l-carnitine and baicalin ameliorated hyperoxia-induced persistent alveolar and vascular simplification. C57BL/6 J mice (< 12 h old) were exposed to 95% (**A**–**C**, **F**, **G**) or 50% (**D**, **E**, **H**) O_2_ for 3 days, and allowed to recover in room air until pnd60. Mice were treated with l-carnitine (LCT, 150 and 300 mg/kg, i.p.) and baicalin (BLN, 50 and 100 mg/kg, i.p.) daily between pnd4 and pnd13. H&E staining was performed to measure Lm and RAC (**A**–**D**), while vWF immunofluorescence was carried out to count vessel numbers in the lung (**F**–**H**). Scale bar: 100 μm in **A** and 50 μm in **F**. N = 5–10. *P < 0.05, **P < 0.01, ***P < 0.001 vs Air/veh (**B**, **C**) or Air (**D**, **E**, **G**, **H**). ^†^*P* < 0.05, ^††^*P* < 0.01, ^†††^*P* < 0.001 vs O_2_/veh using one-way ANOVA followed by Tukey’s multiple comparison test

To further determine the effects of l-carnitine and baicalin on neonatal hyperoxia-induced persistent lung injury, we performed H&E staining and measured Lm and RAC at pnd60 in mice exposed to hyperoxia as neonates. As shown in Fig. 8A–E, neonatal hyperoxia significantly increased Lm and decreased RAC in an oxygen concentration-dependent manner. These effects were attenuated by both baicalin (100 mg/kg) and l-carnitine (300 mg/kg) treatment. In addition, we performed vWF staining and counted the number of pulmonary vessels in hyperoxia-exposed mice treated with l-carnitine and baicalin. As shown in Fig. 8F, G, neonatal hyperoxia at both 50% and 95% O_2_ significantly reduced lung vessel numbers at pnd60. Both l-carnitine and baicalin treatments restored lung vessel numbers in mice exposed to hyperoxia as neonates (Fig. 8F, G). Altogether, l-carnitine and baicalin treatments ameliorate neonatal hyperoxia-induced persistent alveolar and vascular simplification in adult mice.

### Endothelial Cpt1a deletion abolishes the protective effects of l-carnitine and baicalin against neonatal hyperoxia-induced persistent alveolar and vascular simplification

**Fig. 9:**
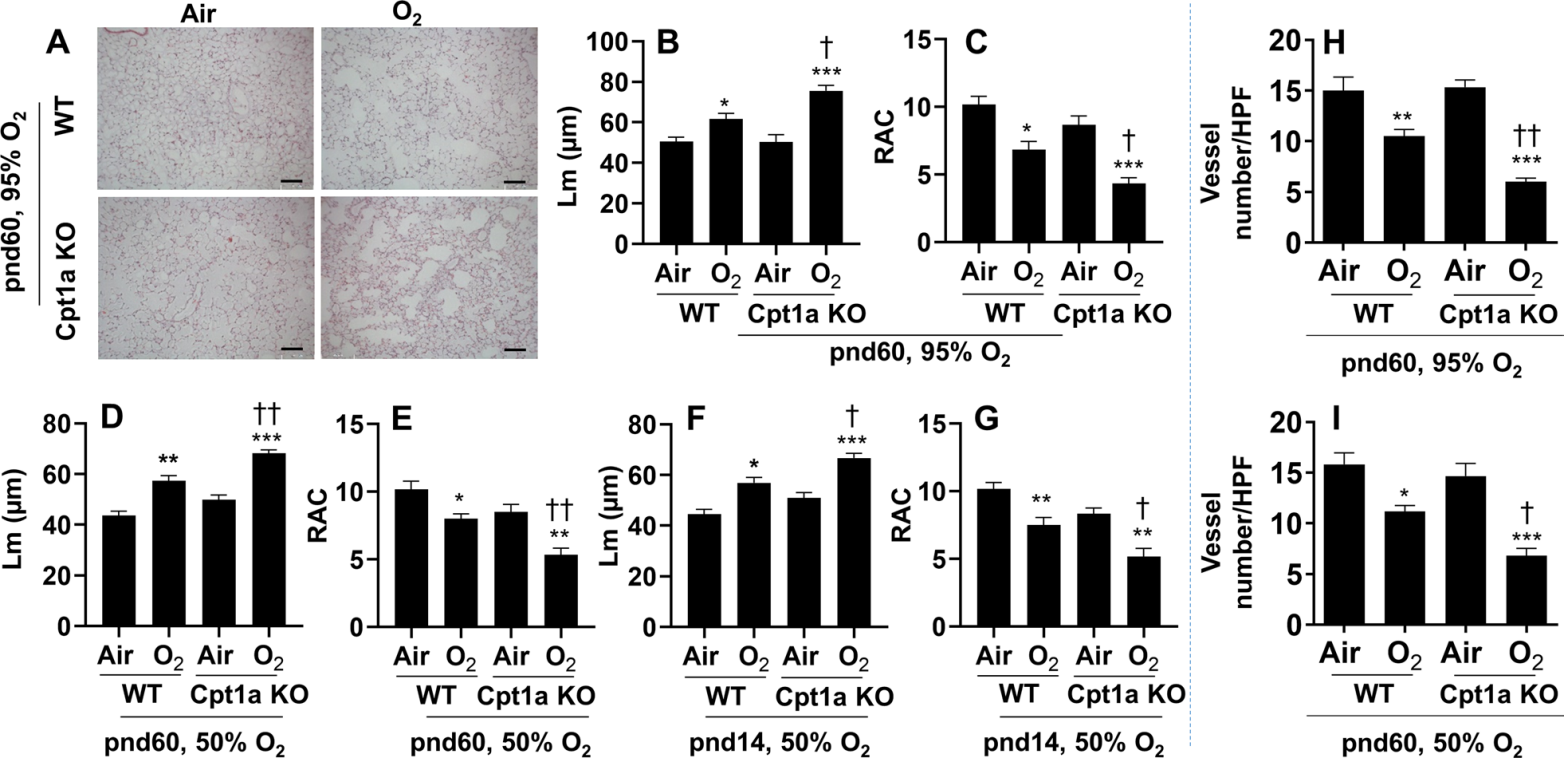
Endothelial cell-specific Cpt1a knockout mice were susceptible to developing hyperoxia-induced persistent alveolar and vascular simplification. Endothelial cell-specific Cpt1a KO mice and WT littermates (< 12 h old) were exposed to 95% (**A**–**C**, **H**) or 50% (**D**–**G**, **I**) O_2_ for 3 days, and allowed to recover in air until pnd14 (**F**, **G**) or pnd60 (**A**–**E**, **H**, **I**). H&E staining was performed to measure Lm and RAC (**A**–**G**), while vWF immunofluorescence was carried out to count vessel numbers in the lung (**H**, **I**). Scale bar: 100 μm. N = 6. *P < 0.05, **P < 0.01, ***P < 0.001 vs corresponding air group. ^†^*P* < 0.05, ^††^*P* < 0.01, ^†††^*P* < 0.001 vs WT/O_2_ using one-way ANOVA followed by Tukey’s multiple comparison test

To determine whether l-carnitine and baicalin inhibits neonatal hyperoxia-induced persistent lung injury via endothelial Cpt1a, we first investigated the susceptibility of endothelial cell-specific Cpt1a knockout mice to developing hyperoxic lung injury. Compared to WT littermates, neonatal hyperoxia further increased Lm and decreased RAC in endothelial cell-specific Cpt1a knockout mice at pnd14 and pnd60 [10] (Fig. 9A–G). In addition, vWF positive vessel numbers were significantly reduced in endothelial cell-specific Cpt1a knockout mice compared to WT littermates exposed to both 50% and 95% O_2_ at pnd60 (Fig. 9H, I). Thus, endothelial cell-specific Cpt1a knockout mice are more susceptible to developing neonatal hyperoxia-induced persistent alveolar and vascular simplification compared to WT littermates.

**Fig. 10:**
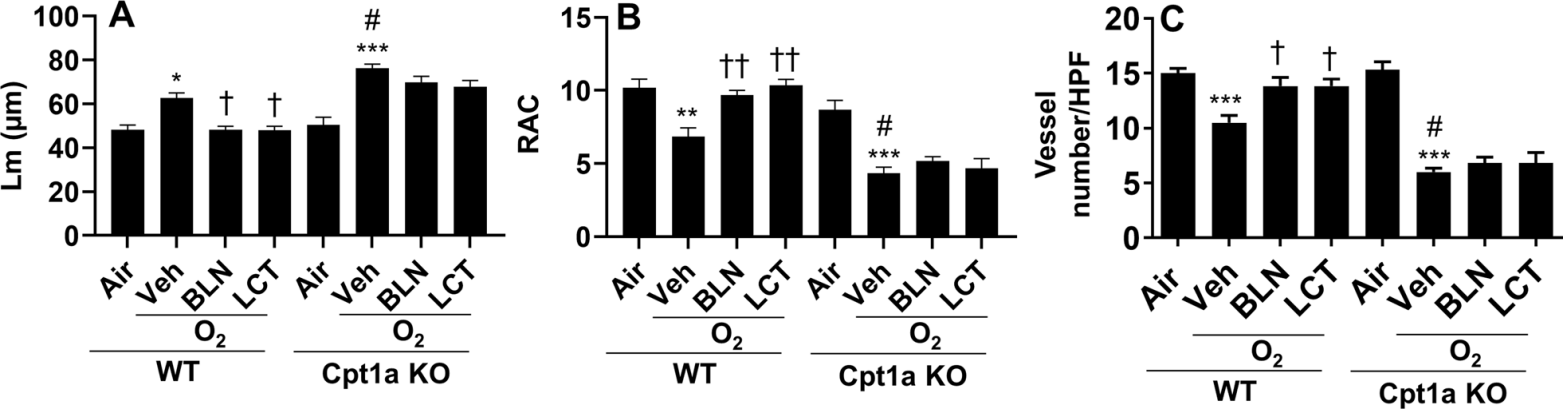
Endothelial Cpt1a deletion diminished the protective effects of l-carnitine or baicalin against hyperoxia-induced persistent alveolar and vascular simplification. Endothelial cell-specific Cpt1a knockout mice and WT littermates (< 12 h old) were exposed to hyperoxia (95% O_2_) for 3 days, and allowed to recover in air until pnd60. Mice were treated with baicalin (BLN, 100 mg/kg, i.p.) and l-carnitine (LCT, 300 mg/kg, i.p.) daily between pnd4 and pnd13. H&E staining was performed to measure Lm and RAC (**A**, **B**), while vWF immunofluorescence was carried out to count vessel numbers in the lung (**C**). N = 6–10. *P < 0.05, **P < 0.01, ***P < 0.001 vs corresponding air group. ^†^*P* < 0.05, ^††^*P* < 0.01 vs corresponding O_2_/Veh. ^#^*P* < 0.05 vs WT/O_2_/Veh using one-way ANOVA followed by Tukey's multiple comparison test

We then treated endothelial cell-specific Cpt1a knockout mice with l-carnitine or baicalin, and investigated whether this led to protection against neonatal hyperoxia-induced persistent lung injury. As expected, both l-carnitine (300 mg/kg) and baicalin (100 mg/kg) significantly decreased Lm and increased RAC at pnd60 in WT mice exposed to hyperoxia (95% O_2_) as neonates (Fig. 10A, B). However, the protective effects of l-carnitine or baicalin against hyperoxia-induced alveolar simplification was abolished in endothelial cell-specific Cpt1a knockout mice. Similarly, endothelial deletion of Cpt1a diminished the protective effects of l-carnitine or baicalin on neonatal hyperoxia-induced vascular simplification at pnd60 (Fig. 10C, D). Altogether, these data demonstrate that l-carnitine or baicalin ameliorates neonatal hyperoxia-induced persistent alveolar and vascular simplification by upregulating Cpt1a in the lung.

## Discussion

Here, we show that upregulating Cpt1a by l-carnitine or baicalin ameliorates neonatal hyperoxia-induced persistent alveolar and vascular simplification in adult mice. This is corroborated by our findings that l-carnitine attenuates hyperoxia-induced alveolar simplification in neonatal mice [10]. These effects are associated with the protection of l-carnitine or baicalin against hyperoxia-induced apoptosis, and impairment of cell migration and angiogenesis in lung endothelial cells. Similar to early reduction of l-carnitine in the lung of hyperoxia-exposed mice [30], premature human infants have lower tissue carnitine stores and decreased blood long-chain acylcarnitines than term infants, particularly in the first two weeks of life [31–34]. Therefore, supplementation of l-carnitine or baicalin could be a potential approach to prevent persistent lung injury in BPD. Indeed, l-carnitine supplementation decreases the duration of mechanical ventilation and the incidence of BPD in premature human infants with respiratory distress syndrome [35].

A previous study reported that Cpt1a-mediated FAO promotes de novo DNA synthesis and increases intermediate products of the tricarboxylic acid cycle, which leads to increased endothelial cell proliferation and vessel sprouting in the retina [16]. This is in contrast with our findings demonstrating that upregulating Cpt1a had no effect on lung endothelial cell proliferation after exposure to hyperoxia. This suggests that the role of Cpt1a in modulating endothelial cell proliferation is vascular bed-specific. BPD is associated with the development of pulmonary hypertension. We and others have observed that hyperoxia followed by air recovery increased lung endothelial cell proliferation, which may cause dysmorphic vascular development, pulmonary vascular remodeling and subsequent pulmonary hypertension [8, 36, 37]. Whether upregulating Cpt1a attenuates neonatal hyperoxia-induced pulmonary vascular remodeling and pulmonary hypertension remains to be investigated. This could not be tested in this study (at pnd60) as we have previously shown that development of pulmonary hypertension after neonatal hyperoxia for 3 days requires a very long interval of time (at pnd120).

Cpt1a and Cpt1b catalyze the transfer of acyl groups from fatty acyl-CoA to carnitine, whereas Cpt1c appears to be involved in the regulation of energy homeostasis independent of acyltransferase activity [38]. Neonatal hyperoxia causes epigenetic changes including DNA and histone methylation, and histone acetylation in the lung [39, 40]. Gene expression of Cpt1 isoforms can be epigenetically regulated through DNA and histone methylation [41–43]. Further study is required to determine whether l-carnitine or baicalin upregulates Cpt1a but not Cpt1b or Cpt1c in lung endothelial cells through epigenetic mechanisms. It has been shown that PPARα and PGC-1α induce Cpt1a gene expression [44]. Additionally, PPARγ is reduced in the lung of rats exposed to hyperoxia for 1 day as neonates [45], and hyperoxic exposure also causes a reduction of PGC-1α in cultured human epithelial cells [46]. Further research is warranted to investigate the effect of l-carnitine on PPARα and PGC-1α in both normoxic and hyperoxic conditions. Under stress, Cpt1 is inactivated in Hela cells treated with hydrogen peroxide [47]. l-carnitine scavenges oxidants, including hydrogen peroxide and superoxide radical, and protects the endogenous antioxidant defense system from peroxidative damage [48, 49]. Thus, l-carnitine may also upregulate Cpt1a by decreasing hyperoxia-induced oxidative stress.

Baicalin is a bioactive natural compound and the main active component of *Scutellaria baicalensis* which has been shown to inhibit oxidative stress, inflammation and apoptosis, as well as modulate angiogenesis [50–52]. This agrees with our findings showing protection of baicalin against apoptosis and impaired angiogenesis in cultured lung endothelial cells exposed to hyperoxia. This may contribute to its protective effects on neonatal hyperoxia-induced alveolar and vascular simplification. Baicalin activates AMPK in adipose tissues and brain [53, 54]. AMPK in turn activates Cpt1a by decreasing its allosteric inhibitor malonyl-CoA through phosphorylation and inactivation of acetyl-CoA carboxylase [55]. Therefore, in addition to directly binding to Cpt1 [23], baicalin may also upregulate Cpt1a by activating AMPK. Indeed, AMPK activity was reduced in the lung of rats exposed to hyperoxia as neonates, and an AMPK activator metformin improves neonatal hyperoxia-induced lung injury [56].

## Conclusions

Both l-carnitine and baicalin attenuate apoptosis, impaired migration and impaired angiogenesis resulting from hyperoxia in cultured lung endothelial cells. These phenotypic changes are associated with increased Cpt1a gene expression and FAO. Neonatal hyperoxia causes persistent alveolar and vascular simplification in an oxygen concentration-dependent manner. Treatment with l-carnitine and baicalin significantly decreases neonatal hyperoxia-induced alveolar and vascular simplification through upregulation of endothelial Cpt1a. Our findings define potential therapeutic approaches using l-carnitine or baicalin to upregulate Cpt1a and prevent persistent alveolar and vascular simplification in premature infants with BPD.

## Data Availability

The datasets used and/or analyzed during the current study are available from the corresponding author on reasonable request.

## Abbreviations

BPD: Bronchopulmonary dysplasia
Cpt1: Carnitine palmitoyltransferase 1
EdU: 5-Ethynyl-2′deoxyuridine
FAO: Fatty acid oxidation
Lm: Mean linear intercept
OCR: Oxygen consumption rate
pnd: Postnatal day
PPARα: Peroxisome proliferator activated receptor α
PGC-1α: PPARγ coactivator-1α
RAC: Radial alveolar count
vWF: Von Wildebrand factor
